# Association Between Vitamin D Levels and Long COVID Signs and Symptoms

**DOI:** 10.3390/medsci13030199

**Published:** 2025-09-18

**Authors:** Karn Matangkha, Vichit Punyahotara, Jarasphol Rintra, Phakkharawat Sittiprapaporn

**Affiliations:** 1 Department of Anti-Aging and Regenerative Medicine, School of Anti-Aging and Regenerative Medicine, Mae Fah Luang University, Bangkok 10110, Thailand; mdkarn.m@gmail.com (K.M.); vichit.pun@mfu.ac.th (V.P.); jarasphol.rin@mfu.ac.th (J.R.); 2 Department of Anti-Aging and Regenerative Science, School of Anti-Aging and Regenerative Medicine, Mae Fah Luang University, Bangkok 10110, Thailand; 3 MAS Neuroscience Center, School of Anti-Aging and Regenerative Medicine, Mae Fah Luang University, Bangkok 10110, Thailand

**Keywords:** long COVID, COVID-19, post-COVID-19 signs and symptoms, vitamin D

## Abstract

Background: “Long COVID” refers to a condition in which individuals continue to experience persistent signs and symptoms even after recovering from the initial COVID-19 infection. Signs and symptoms that persist can affect multiple organs in the body. Vitamin D is an essential nutrient that plays a crucial role, particularly in the immune system, and may be linked to the development of long COVID. Objective: The study aimed to investigate the association between vitamin D levels and the prevalence of long COVID signs and symptoms in COVID-19 patients. Materials and Methods: The study enrolled 170 COVID-19 patients with mild signs and symptoms and confirmed COVID-Ag or RT-PCR tests. The subjects were aged 18–59 years. All patients had 25(OH)D levels measured within 60 days of COVID-19 diagnosis and had been followed for at least 3 months post-infection. Data collected included demographic characteristics, serum 25(OH)D levels, and self-reported long COVID signs and symptoms questionnaire responses. Results: The study results indicated a female-to-male ratio of 1.1:1 and a mean age of 45.87 ± 8.65 years; of these, 62.4% received three doses of the COVID-19 vaccine, and 64.7% developed long COVID. The most prevalent signs and symptoms were respiratory (55.3%), skin (50.6%), and general (39.4%). The median blood vitamin D level was 22.96 ng/mL, with 41.2% of subjects having insufficient levels, 30.6% having deficient levels, and 28.2% having sufficient levels. Patients with long COVID had significantly lower vitamin D levels compared with those without long COVID (21.52 ng/mL vs. 25.46 ng/mL; *p* < 0.05). Multivariable analysis found that vitamin D deficiency was significantly associated with overall long COVID signs and symptoms (Adj. OR, 5.80 [95% CI: 2.10, 16.13]). Additionally, vitamin D deficiency significantly increased the number of long COVID systemic signs and symptoms (Adj. IRR, 3.30 [2.12, 5.12]). Conclusion: Assessing and maintaining vitamin D levels, vitamin D supplementation, and sunlight exposure in COVID-19 patients can reduce the risk and severity of long-term COVID-19 signs and symptoms.

## 1. Introduction

Coronavirus disease 2019 (COVID-19), caused by the novel coronavirus SARS-CoV-2, was first identified in Wuhan, China, in December 2019. Since then, it has spread globally, significantly impacting health, economies, and societies [1]. Common signs and symptoms include fever, dry cough, fatigue, and the production of sputum [2]. While most people experience mild signs and symptoms, elderly people and those with underlying health conditions may develop more severe signs and symptoms. Control and prevention measures include screening, mask-wearing, handwashing, social distancing, quarantine, and vaccination. In 2022, Thailand declared COVID-19 an endemic disease and eased its preventive measures [3].

Long COVID, also known as Post-Acute Sequelae of SARS-CoV-2 infection (PASC), refers to a condition where patients continue to experience persistent signs and symptoms after recovering from the acute phase of COVID-19. These signs and symptoms last for at least 12 weeks following the initial infection. Both the World Health Organization (WHO) and the National Institute for Health and Care Excellence (NICE) have issued various definitions of this condition [4,5].

Long COVID occurs in approximately 10–30% of COVID-19 patients [6]. A survey on the incidence of long COVID in patients with a history of COVID-19 in the United States found rates of 18.9% and 11.0% in 2022 and 2023, respectively. The condition was more commonly observed in individuals aged 60 and older [7]. In Thailand, 29.9% of patients have reported signs and symptoms consistent with long COVID [8]. A meta-analysis conducted in 2024 reported a global prevalence of post-COVID-19 syndrome of approximately 41.79% (95% confidence interval [CI]: 39.70–43.88%, I^2^ = 51%, *p* = 0.03) [9]. More than 200 signs and symptoms have been identified, affecting various body systems [10]. These include general signs and symptoms such as fatigue and fever; respiratory signs and symptoms such as difficulty breathing and coughing; cardiovascular issues such as heart palpitations and chest pain; neurological signs and symptoms such as loss of smell, loss of taste, and headaches; gastrointestinal signs and symptoms such as diarrhea and nausea; dermatological signs and symptoms such as rashes and hair loss; musculoskeletal signs and symptoms such as muscle pain and joint pain; and psychiatric signs and symptoms such as anxiety and depression [6]. Risk factors for developing long COVID include being female, over the age of 50, working in the transportation sector, smoking, having an underlying disease, experiencing more than five signs and symptoms during the acute phase of COVID-19, and having a severe form of the disease [11,12]. Additionally, abnormal blood test results and deficiencies in vitamins and minerals have also been associated with a higher risk of developing long COVID [13]. A community-based study in Rio de Janeiro further confirmed risk factors, including age and comorbidities [14]. Recent evidence also highlighted that long COVID subtypes remain clinically significant up to 2 years post-infection [15].

Serological markers of prior infection are important in contextualizing long COVID. Anti-nucleocapsid (anti-N) antibodies are generated only by natural SARS-CoV-2 infection, not by vaccines targeting the spike protein. They therefore serve as useful markers for identifying prior infection, especially in settings with high vaccination coverage. Anti-N seropositivity has been shown to remain detectable for many months post infection, although it declines over time [16,17].

Vitamin D is an essential vitamin that plays a key role in supporting various aspects of health, particularly in regulating the immune system’s response. It enhances both innate and adaptive immunity [18]. Studies have shown that vitamin D levels in the blood are associated with the severity of COVID-19. Individuals with sufficient (>50 nmol/L) and insufficient (25–50 nmol/L) vitamin D levels are approximately 50% less likely to develop severe signs and symptoms of COVID-19 compared with individuals who are deficient in vitamin D (<25 nmol/L) [19]. It is clear that low blood levels of vitamin D impact the severity of COVID-19, increasing mortality rates, ICU admissions, length of hospital stays, and the need for ventilators; moreover, evidence from 2024 further emphasizes the therapeutic role of vitamin D supplementation in COVID-19 patients, showing reduced ICU admissions and mortality [20,21].

The severity of COVID-19 also influences the likelihood of developing long COVID. Patients with severe and critical COVID-19 infection are more likely to develop long COVID compared with those with mild signs and symptoms [11]. Recent studies have suggested that low levels of vitamin D may not only increase the severity of acute COVID-19 but also contribute to long COVID; however, evidence regarding the association between vitamin D and the symptoms and signs of long COVID remains inconclusive. Further studies are needed to explore the association between vitamin D levels and long COVID signs and symptoms, specifically to determine whether low blood levels of vitamin D increase the risk of long COVID in patients who have recovered from COVID-19. This would involve evaluating signs and symptoms across eight body systems associated with long COVID three months after recovery. The findings from such research could provide insights into the role of vitamin D deficiency or insufficiency in long COVID and help establish a potential relationship between vitamin D deficiency and the condition. Understanding this connection is critical, as it could lead to preventive strategies, including supplementation and lifestyle modifications, aimed at reducing the incidence and severity of long COVID in populations at risk.

## 2. Materials and Methods

This was a descriptive cross-sectional study approved by the Research Ethics Committee of Mae Fah Luang University (COA: 166/2024, EC 24104-20, approval date: 13 August 2024). Participants were COVID-19 patients whose diagnoses were confirmed through COVID-Ag or RT-PCR tests. The inclusion criteria required participants to have had mild signs and symptoms, be aged 18–59, have 25(OH)D results obtained within two months of infection, and have received treatment at a clinic in Thailand with follow-up more than 12 weeks after the index infection for long COVID assessment. Exclusion criteria included any comorbidities and a history of vitamin D supplementation; therefore, all participants had no comorbidities.

The sample size was calculated using the formula for comparing two independent means, based on the research by Di Fillppo et al. [18], with parameters set at α = 0.05 and β = 0.20. The sample size was adjusted by 10%, resulting in 170 participants.

The data collection tools were divided into three parts: demographic data, 25(OH)D levels, and a self-reported long COVID signs and symptoms questionnaire. The demographic data included details such as gender, age, body mass index, vaccination status, and ethnicity (all participants were Thai). Long COVID signs and symptoms were categorized into eight systems: general (e.g., fatigue, fever); respiratory (e.g., dyspnea, cough); cardiovascular (e.g., palpitations, tachycardia, chest pain); neurological (e.g., loss of taste or smell, headaches, dizziness); gastrointestinal (e.g., dizziness, diarrhea, stomach pain); musculoskeletal (e.g., muscle, joint, and bone pain); skin-related (e.g., rash, hair loss); and psychiatric (e.g., anxiety, depression, sleep disorders). The occurrence of long COVID signs and symptoms was defined as the presence of signs and symptoms in any one of the eight systems at least 12 weeks after the initial infection and was classified dichotomously (either with or without the presence of that symptom). The self-reported long COVID signs and symptoms questionnaire was adapted from the Long-Term Health Impact of COVID-19 Patients Survey by the Department of Medical Services, Ministry of Public Health (the Kuder–Richardson coefficient of reliability (KR-20) was 0.8638) [22]. Formal validations in broader Thai populations have not been established.

**Figure 1:**
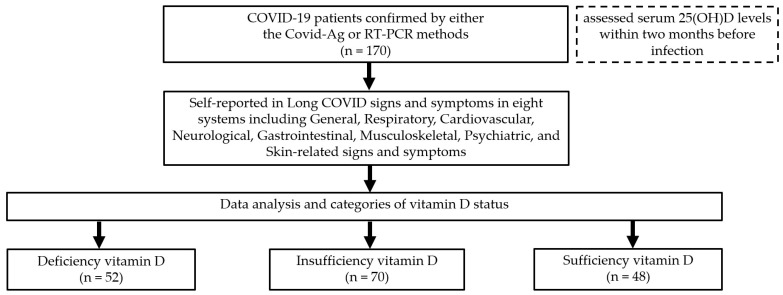
Study flow and categorization of vitamin D status in relation to long COVID signs and symptoms in COVID-19 patients.

Serum vitamin D status was assessed using 25-hydroxy vitamin D (25(OH)D) levels obtained from clinic electronic records. Samples (serum or plasma heparinized with sodium heparin) were analyzed using Chemiluminescent Microparticle Immunoassay (CMIA). Results were reported in ng/mL and classified according to the Endocrine Society’s criteria as deficiency (<20 ng/mL), insufficiency (20–30 ng/mL), and sufficiency (>30 ng/mL) [23]. Because none of the participants had serum 25(OH)D levels < 5 ng/mL, a severely deficient category was not included. The flow of the study is presented in Figure 1.

Descriptive statistics were used to present demographic data and self-reported long COVID signs and symptoms, expressed as means with standard deviations or medians with interquartile ranges for continuous variables, and frequencies and percentages for categorical variables. Demographic data among the three vitamin D status groups were compared using one-way ANOVA for continuous variables and the chi-square test for categorical variables. The association between vitamin D levels and the occurrence of long COVID signs and symptoms was measured using the Mann–Whitney U test. Multivariable analysis of the vitamin D level factor affecting the occurrence of long COVID signs and symptoms was conducted using multiple logistic regression, presenting adjusted odds ratios (ORs) and 95% confidence intervals (CIs). The enter method was applied, including all predefined covariates simultaneously (sex, age, BMI, number of COVID-19 vaccine doses, duration from vitamin D measurement to infection, and duration of infection to long COVID self-report). Vitamin D sufficiency served as the reference category. 

Additionally, factors of vitamin D level affecting the number of long COVID systemic signs and symptoms were examined using negative binomial regression, with incident rate ratios (IRRs) and 95% CIs reported. The data were analyzed using STATA (StataCorp. Stata Statistical Software: Release 18. College Station, TX, USA: StataCorp LLC; 2023). All statistical tests were two-tailed, and the level of statistical significance was set at 0.05 (α = 0.05).

## 3. Results

A total of 170 COVID-19 patients, comprising a similar number of males and females, were enrolled in the study. The mean age was 45.87 ± 8.65 years, and the body mass index was 24.90 ± 4.72 kg/m^2^. Most (62.1%) were overweight. The majority (62.4%) had received three doses of the COVID-19 vaccine. The median vitamin D level was 22.96 ng/mL (IQR 28.77, 31.7). The majority (41.2%) had insufficient vitamin D levels (20–30 ng/mL), followed by vitamin D deficiency (<20 ng/mL) at 30.6%, and vitamin D sufficiency (>30–100 ng/mL) at 28.2%. Comparison of demographic data across the three vitamin D status groups showed no statistically significant differences for any variable (*p* > 0.05) (Table 1).

The majority of patients (64.7%) had long-term COVID signs and symptoms. When categorized by body system, respiratory signs and symptoms were the most common at 55.3%, followed by skin signs and symptoms at 50.6%, general signs and symptoms (fatigue and fever) at 39.4%, and mental signs and symptoms at 30.0%. Among those with long COVID signs and symptoms, 24.1% had 1–2 signs and symptoms, 18.8% had 3–4 signs and symptoms, 13.5% had 5–6 signs and symptoms, and 8.3% had 7–8 signs and symptoms. Comparison of vitamin D levels between those with and without long COVID signs and symptoms revealed that those with long COVID signs and symptoms had a median vitamin D level that was lower than those without long COVID signs and symptoms at a statistically significant level of 0.05 (21.52 and 25.46 ng/mL; *p* < 0.001). Similarly, when classified by body systems, those with long COVID signs and symptoms consistently had significantly lower vitamin D levels compared with those without long COVID signs and symptoms (*p* < 0.05) (Table 2).

**Figure 2:**
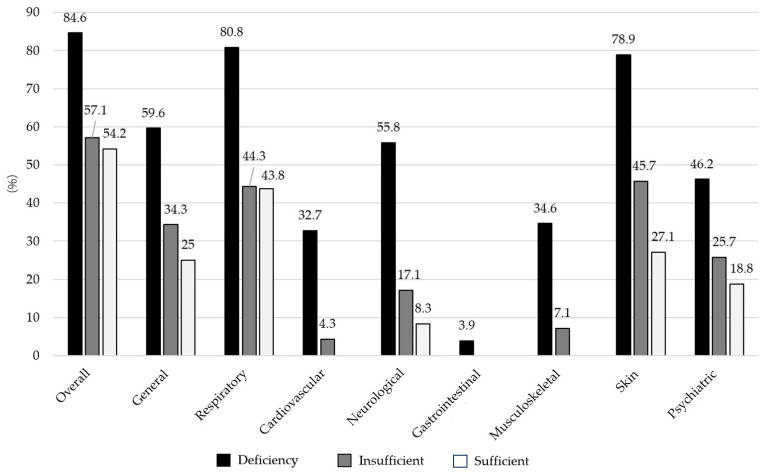
Prevalence of eight clusters of long COVID signs and symptoms based on vitamin D status.

The prevalence of long COVID signs and symptoms was highest among those with vitamin D deficiency (59.6%), followed by vitamin D insufficiency (34.3%) and vitamin D sufficiency (25.0%). Similarly, the prevalence of long COVID signs and symptoms was highest (78.9–80.8%) in those with vitamin D deficiency, particularly for respiratory and skin signs and symptoms (Figure 2).

A multivariable analysis of factors influencing long COVID signs and symptoms revealed that vitamin D deficiency had a statistically significant impact on long COVID signs and symptoms at the 0.05 level (Adj. OR, 5.80 [95% CI: 2.10, 16.01]; *p* = 0.001); in other words, patients with vitamin D deficiency were 5.80 times more likely to experience long COVID signs and symptoms compared with patients with sufficient vitamin D levels. When categorized by body system, vitamin D deficiency significantly influenced the occurrence of long COVID signs and symptoms in various body systems, with statistically significant results at the 0.05 level; these included general signs and symptoms (Adj. OR, 4.55 [95% CI: 1.88, 10.87]; *p* = 0.001), respiratory signs and symptoms (Adj. OR, 6.06 [95% CI: 2.37, 15.54]; *p* < 0.001), cardiovascular signs and symptoms (Adj. OR, 22.73 [95% CI: 5.88, 87.14]; *p* < 0.001), neurological signs and symptoms (Adj. OR, 16.22 [95% CI: 4.81, 54.65]; *p* < 0.001), musculoskeletal signs and symptoms (Adj. OR, 13.77 [95% CI: 4.54, 41.82]; *p* < 0.001), skin signs and symptoms (Adj. OR, 11.28 [95% CI: 4.30, 29.57]; *p* < 0.001), and psychiatric signs and symptoms (Adj. OR, 3.97 [95% CI: 1.56, 10.08]; *p* = 0.004) (Table 3).

**Figure 3:**
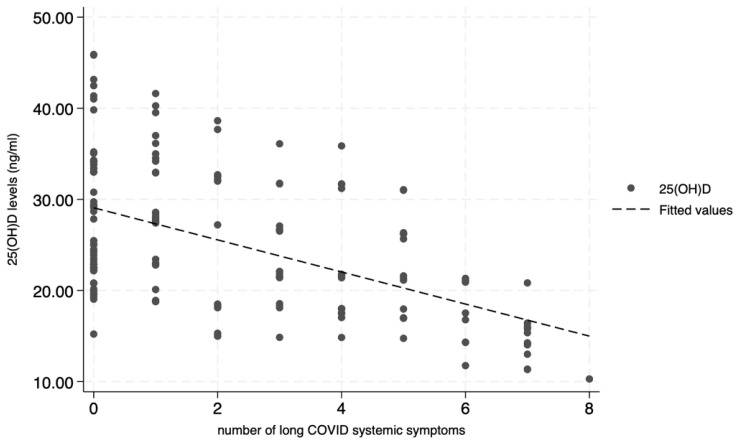
Scatter plot of the correlation between vitamin D levels and the number of long COVID systemic signs and symptoms.

A multivariable analysis of factors influencing long COVID signs and symptoms revealed that vitamin D deficiency had a statistically significant impact on the number of long COVID systemic signs and symptoms (*p* < 0.001). Patients with vitamin D deficiency showed 3.3 times more long COVID systemic signs and symptoms compared with those with sufficient vitamin D levels (IRR, 3.30 [95% CI: 2.12, 5.12]; *p* < 0.001). Meanwhile, individuals with insufficient vitamin D levels had 1.54 times more long COVID systemic signs and symptoms compared with those with sufficient vitamin D levels; however, this difference was not statistically significant (IRR, 1.54 [95% CI: 0.99, 2.40]; *p* = 0.057). The mean systemic number of long COVID signs and symptoms was 3.92 for those with vitamin D deficiency, 1.8 for those with insufficient vitamin D levels, and 1.22 for those with sufficient vitamin D levels (Table 4). The scatter plot showing the association between vitamin D levels and the number of long COVID systemic signs and symptoms reveals a negative correlation; in other words, lower vitamin D levels are associated with a higher number of long COVID systemic signs and symptoms, whereas higher vitamin D levels are correlated with fewer long COVID signs and symptoms (Figure 3).

## 4. Discussion

This study found a prevalence of 64.74% of long COVID among COVID-19 patients, with the most common signs and symptoms being respiratory (55.3%), skin-related (50.6%), and general (39.4%). The most frequently reported specific signs and symptoms were cough (51.8%), hair loss (47.7%), and fatigue (39.4%). Long COVID refers to the condition where patients, after recovering from COVID-19, continue to experience persistent signs and symptoms across various body systems. These signs and symptoms cannot be explained by other diagnoses and may be caused by viral genome fragments or viral antigens that no longer contribute to infection but continue to affect the immune system, leading to inflammation in different parts of the body [24].

The prevalence of long COVID in this study is comparable to that in another hospital-based cross-sectional study conducted in Thailand, which reported a rate of 64.9% [25]. However, it is higher than those of cross-sectional community surveys, which reported rates of 29.9% [8] and 40.5% [26]. Research studies conducted in different regions show varied prevalence rates, including 40.7% in a population-based cohort study in the UK [27], 29.6% in Brazil’s community-based cohort study [12], and 90.4% in China [11]. Meta-analyses and systematic reviews have found an overall prevalence ranging from 43.0% to 56.9% (systematic reviews and meta-analyses across regions) [28,29], with regional variations: 44.0% in Europe, 51.0% in Asia, and 31.0% in the Americas. Common symptoms reported across studies tend to include general and respiratory signs and symptoms. Variations in the prevalence of signs and symptoms may be due to differences in study methods, populations, and follow-up periods.

The median vitamin D level among the COVID-19 patients in this study was 22.96 ng/mL. Most participants had insufficient vitamin D levels (20–30 ng/mL) (41.2%), 30.6% were vitamin D deficient (<20 ng/mL), and the remaining 28.2% had sufficient vitamin D levels (>30–100 ng/mL). Since the participants lived in Bangkok, their low vitamin D levels may be attributed to several urban living factors, such as spending time indoors, working in buildings with limited sunlight exposure, high levels of air pollution that block sunlight, the use of sunscreen (which reduces vitamin D production from sunlight), low dietary intake of vitamin D-rich foods, and the high population density, which limits access to sunny or outdoor areas. These factors collectively contribute to the insufficient vitamin D levels in Bangkok residents. This finding is consistent with the study by Chailurkit et al. [30] conducted between 2019 and 2020, which found that people living in Bangkok, or the Central Region, had lower vitamin D levels compared with those living in other areas in Thailand. Overall, 31.0% of the Thai population had insufficient (<30 ng/mL) vitamin D levels [30].

This study demonstrated a significant association between vitamin D levels and the development of long COVID. It found that individuals with long COVID signs and symptoms had significantly lower blood vitamin D levels compared with those without long COVID signs and symptoms (21.52 ng/mL vs. 25.46 ng/mL, respectively) across both the overall and each of the eight systemic signs and symptoms. Additionally, those with vitamin D deficiency were 5.80 times more likely to develop long COVID signs and symptoms and 3.3 times more likely to experience multiple long COVID systemic signs and symptoms compared with those with sufficient vitamin D levels. Among those deficient in vitamin D, the prevalence of long COVID signs and symptoms was as high as 84.6%, with respiratory signs and symptoms reported by 80.8% of patients, skin-related signs and symptoms reported by 78.9% of patients, and general signs and symptoms reported by 59.6% of patients. These findings highlight the association between low vitamin D levels and the prevalence of long COVID signs and symptoms.

Because individuals with a history of vitamin D supplementation were excluded and no supplementation was provided during follow-up, our study did not address the reversibility of long COVID signs and symptoms after vitamin D supplementation.

Vitamin D plays a crucial role in supporting the immune system and the body’s response to COVID-19 infections and post-infection complications by reducing inflammation. Sufficient vitamin D levels have been associated with reduced severity of COVID-19 at onset, as it enhances immune responses and attenuates cytokine storms [31,32], which may contribute to lowering the risk of long-term complications.

Vitamin D has been recognized as a potent immunomodulator that enhances the activity of the innate immune system, such as monocytes and macrophages, while also helping to maintain immune tolerance and reduce the risk of autoimmune reactions [33]. In addition, its anti-inflammatory properties are widely documented, including the ability to suppress pro-inflammatory cytokine production and regulate systemic immune responses, thereby contributing to improved health outcomes [34]. Moreover, vitamin D helps maintain the integrity of epithelial cells in the respiratory system [35], playing a protective role against viral invasion and potentially reducing the severity of infections. It also supports overall health, including bone and muscle function and mental well-being, all of which can promote faster recovery from infections and reduce chronic symptoms associated with long COVID [36]. There is also evidence that low vitamin D levels are associated with long COVID, and that combined vitamin D and magnesium supplementation may help alleviate persistent signs and symptoms [37]. In addition to its immunomodulatory and anti-inflammatory actions, vitamin D sufficiency has also been implicated in multiple cardio-metabolic pathways. Adequate vitamin D status has been shown to attenuate vascular inflammation and oxidative stress, enhance endothelial nitric oxide (NO) bioavailability, suppress the renin–angiotensin–aldosterone system, and inhibit vascular smooth muscle cell proliferation. Clinically, vitamin D sufficiency has been associated with lower arterial stiffness and blood pressure, improvements in lipid profiles, and better glycemic control via effects on pancreatic β-cell function and insulin sensitivity [38].

The observational study conducted by Nielsen NM et al. [19] showed that individuals with sufficient or insufficient vitamin D levels had a 50% lower risk of severe COVID-19 compared with individuals with vitamin D deficiency. Similarly, the retrospective matched study conducted by Di Filippo et al. [18] found that vitamin D levels in patients with long COVID were significantly lower than in patients without long COVID (20.1 ng/mL vs. 23.2 ng/mL, *p* = 0.030), with low vitamin D levels increasing the risk of long COVID by 1.09 times. Another prospective case-control study conducted by Cardoso F et al. [39] reported that the mean vitamin D level in patients with severe COVID-19 pneumonia was 26.8 ± 7.6 ng/mL, which was lower than the control group’s mean of 28.6 ± 7.4 ng/mL. Patients with vitamin D deficiency were three times more likely to develop severe COVID-19 pneumonia (95% CI: 1.79, 5.10); this result was statistically significant.

The findings on vitamin D deficiency contributing to the number of long COVID signs and symptoms across multiple systems are consistent with the study conducted by Guerrero-Romero et al. [40] who found that individuals with hypomagnesemia (≤1.8 mg/dL) and vitamin D deficiency (<30 ng/mL) had 3.1 times more clinical signs and symptoms associated with long COVID compared with the control group. The common signs and symptoms in these groups included fatigue, memory loss, attention disorders, joint pain, anxiety, sleep disorders, myalgia, and depression [40].

Based on the findings of this study, it is recommended that vitamin D levels be monitored in COVID-19 patients and at-risk groups, particularly the elderly and individuals with underlying conditions and obesity, to prevent and reduce the risk of long COVID. Promoting sunlight exposure and vitamin D supplementation both before and after infection is crucial for enhancing immune function, minimizing the severity of signs and symptoms, and facilitating faster recovery. Public health agencies should raise awareness about the benefits of vitamin D and disseminate the findings of this research to the public and relevant stakeholders to support effective treatment and rehabilitation planning for COVID-19 patients.

This study has some limitations. First, the relatively small sample size and the use of self-reported questionnaires may introduce response bias. The questionnaire has not been fully validated in Thai populations, which may limit generalizability. Some odds ratios were very high (e.g., OR > 20), which may reflect model instability due to sparse data. In addition, vitamin D levels were measured up to 60 days before infection, which may not accurately represent the status at the time of infection, because 25(OH)D can vary with season, sunlight exposure, dietary intake, or intercurrent illness; this temporal misclassification could lead to an underestimation of the observed associations. Serum vitamin D levels were not reassessed after recovery from COVID-19; therefore, changes in vitamin D status during the post-infection period could not be evaluated.

Given these limitations, future long-term studies should be conducted to assess changes in vitamin D levels and long COVID signs and symptoms over time. In-depth research into the biological mechanisms linking vitamin D levels to signs and symptoms across multiple body systems, such as the nervous, muscular, and cardiovascular systems, is also required. Utilizing standardized medical tools and internationally recognized questionnaires will ensure the collection of detailed and reliable data. Finally, factors such as physical activity, nutrition, and sunlight exposure should be further studied to accurately assess the effects of vitamin D on long COVID.

## 5. Conclusions

This study highlights a significant association between vitamin D levels and the prevalence and severity of long COVID signs and symptoms across multiple body systems. Patients with vitamin D deficiency were significantly more likely to experience long COVID signs and symptoms, with an increased risk of experiencing systemic signs and symptoms. These findings suggest the importance of monitoring and maintaining sufficient vitamin D levels in COVID-19 patients to potentially reduce the risk of long COVID, particularly through supplementing and promoting sunlight exposure. Public health initiatives might raise awareness of the benefits of vitamin D, especially for at-risk populations, such as elderly individuals and those with underlying diseases and obesity. Further research is warranted to explore the underlying biological mechanisms and long-term effects of vitamin D on COVID-19 recovery.

## Figures and Tables

**Table 1:** Demographic and COVID-19 vaccination data according vitamin D status.

	Total (*n* = 170)	Vitamin D Status			*p*-Value
		Deficiency (*n* = 52, 30.6%)	Insufficiency (*n* = 70, 41.2%)	Sufficiency (*n* = 48, 28.2%)	
Sex, n (%)					
Male	83 (48.8)	26 (50.0)	34 (48.6)	23 (47.9)	0.977
Female	87 (51.2)	26 (50.0)	36 (51.4)	25 (52.1)	
Age (years), mean ± SD	45.87 ± 8.65	45.85 ± 8.87	45.17 ± 8.85	46.92 ± 8.18	0.563
BMI (kg/m^2^), mean ± SD	24.90 ± 4.72	24.87 ± 4.55	24.48 ± 4.67	25.54 ± 5.01	0.489
<18.5	17 (10.1)	4 (7.8)	8 (11.4)	5 (10.4)	
18.5–22.9	47 (27.8)	16 (31.4)	20 (28.6)	11 (22.9)	0.866
≥23.0	105 (62.1)	31 (60.8)	42 (60.0)	32 (66.7)	
Number of COVID-19 vaccine doses, n (%)					
1 dose	11 (6.5)	4 (7.7)	4 (5.7)	3 (6.3)	0.911
2 doses	30 (17.7)	9 (17.3)	12 (17.1)	9 (18.8)	
3 doses	106 (62.4)	30 (57.7)	44 (62.9)	32 (66.7)	
4 doses	23 (13.5)	9 (17.3)	10 (14.3)	4 (8.3)	
Total 25(OH)D level (ng/mL), median (IQR)	22.96 (18.77, 31.7)	16.98 (14.91, 18.35)	23.34 (21.6, 26.7)	34.15 (32.6, 37.34)	

Data were analyzed with the Chi-square test and one-way ANOVA.

**Table 2:** Comparison of vitamin D levels between individuals with and without long COVID signs and symptoms, classified by affected signs and symptoms.

Long COVID Signs and Symptoms	Yes		No		*p*-Value
	n (%)	Median (IQR)	n (%)	Median (IQR)	
Overall ^#^	110 (64.7)	21.52 (17.5, 28.48)	60 (35.3)	25.46 (22.42, 33.84)	<0.001 *
General	67 (39.4)	21.21 (16.36, 26.51)	103 (60.6)	25.46 (20.82, 33.0)	<0.001 *
Respiratory	94 (55.3)	21.32 (16.96, 27.61)	76 (44.7)	26.65 (22.48, 33.99)	<0.001 *
Cardiovascular	20 (11.8)	15.33 (14.08, 16.39)	150 (88.2)	25.28 (20.77, 32.5)	<0.001 *
Neurological	45 (26.5)	17.03 (14.84, 21.31)	125 (73.5)	26.67 (21.52, 32.97)	<0.001 *
Gastrointestinal	2 (1.2)	12.27 (10.28, 14.25)	168 (98.8)	23.12 (18.85, 31.7)	0.002 *
Musculoskeletal	23 (13.5)	16.36 (14.03, 18.9)	147 (86.5)	25.46 (20.8, 32.5)	<0.001 *
Skin	86 (50.6)	21.0 (16.42, 26.34)	84 (49.4)	27.82 (22.42, 34.23)	<0.001 *
Psychiatric	51 (30.0)	20.91 (15.36, 26.2)	119 (70.0)	25.46 (20.16, 33.0)	<0.001 *

Data were analyzed with the Mann–Whitney U test. **^#^** Overall: Had symptoms in any system. * Statistically significant at the 0.05 level (α = 0.05).

**Table 3:** Multivariable analysis of vitamin D status associated with long COVID signs and symptoms.

Long COVID Signs and Symptoms	Vitamin D Deficiency		Vitamin D Insufficiency	
	Adjusted OR (95% CI) ^¥^	*p*-Value	Adjusted OR (95% CI) ^¥^	*p*-Value
Overall ^#^	5.80 (2.10, 16.01)	0.001 *	1.25 (0.57, 2.74)	0.576
General	4.55 (1.88, 10.87)	0.001 *	1.65 (0.72, 3.79)	0.239
Respiratory	6.06 (2.37, 15.54)	<0.001 *	1.10 (0.51, 2.37)	0.807
Cardiovascular ^‡^	22.63 (5.88, 87.14)	<0.001 *	(Combined with sufficiency group)	
Neurological	16.22 (4.81, 54.65)	<0.001 *	2.49 (0.74, 8.43)	0.142
Gastrointestinal	N/A ^†^	-		
Musculoskeletal ^‡^	13.77 (4.54, 41.82)	<0.001 *	(Combined with sufficiency group)	
Skin	11.28 (4.30, 29.57)	<0.001 *	2.44 (1.08, 5.50)	0.032 *
Psychiatric	3.97 (1.56, 10.08)	0.004 *	1.51 (0.61, 3.76)	0.335
Reference group: Vitamin D sufficiency status				

Data were analyzed with multiple logistic regression (enter method). Vitamin D sufficiency was used as the reference category. ^#^ Overall: Had symptoms in any system. ^¥^ Adjusted for sex, age, BMI, number of COVID-19 vaccine doses, duration of vitamin D measurement to infection, and duration of infection to long COVID self-report. ^‡^ Reference group combines insufficiency and sufficiency groups because the frequency is 0 in the cross-tabulation table. ^†^ Adjusted odds ratio does not appear because the frequency is 0 in the cross-tabulation table. * Statistically significant at the 0.05 level (α = 0.05).

**Table 4:** Multivariable analysis of vitamin D status associated with the number of long COVID systemic signs and symptoms.

Vitamin D Status	Adjusted IRR (95%CI) ^¥^	Margin (95%CI)	*p*-Value
Deficiency	3.30 (2.12, 5.12)	3.92 (2.89, 4.95)	<0.001 *
Insufficiency	1.54 (0.99, 2.40)	1.80 (1.33, 2.28)	0.057
Sufficiency	Reference	1.22 (0.79, 1.64)	

IRR: incidence rate ratio. Data were analyzed with negative binomial regression. ^¥^ Adjusted for sex, age, BMI, number of COVID-19 vaccine doses, duration of vitamin D measurement to infection, and duration of infection to long COVID self-report. * Statistically significant at the 0.05 level (α = 0.05).

## Data Availability

The original contributions presented in this study are included in the article. Further inquiries can be directed to the corresponding author.

## References

[1] Faculty of Medicine, Ramathibodi Hospital (2020). Basic Knowledge of COVID-19, Part 1.

[2] Thai Health Promotion Foundation (2020). Fighting COVID-19 Together: A Self-Care Guide for the Public.

[3] National Health Security Office (NHSO) (2022). Cancellation of Reasonable Grounds Related to COVID-19 to Support Its Endemic Status. R. Gaz..

[4] World Health Organization (2021). A Clinical Case Definition of Post COVID-19 Condition by a Delphi Consensus. https://apps.who.int/iris/handle/10665/345824.

[5] (2024). COVID-19 Rapid Guideline: Managing the Long-Term Effects of COVID-19.

[6] Kokolevich Z.M., Crowe M., Mendez D., Biros E., Reznik J.E. (2022). Most Common Long COVID Physical Symptoms in Working Age Adults Who Experienced Mild COVID-19 Infection: A Scoping Review. Healthcare.

[7] Ford N.D., Slaughter D., Edwards D., Dalton A., Perrine C., Vahratian A., Dietz P., Prue C., Kompaniyets L., Ashley C. (2023). Long COVID and Significant Activity Limitation Among Adults, by Age-United States, 1–13 June 2022 to 7–19 June 2023. MMWR Morb. Mortal. Wkly. Rep..

[8] Wongsoemsin S., Chinoraso J., Yeekian C. (2022). Symptom and Factors Affecting Severity of Long COVID. Chonburi Hosp. J..

[9] Sk Abd Razak R., Ismail A., Abdul Aziz A.F., Suddin L.S., Azzeri A., Sha’ari N.I. (2024). Post-COVID syndrome prevalence: A systematic review and meta-analysis. BMC Public Health.

[10] Davis H.E., McCorkell L., Vogel J.M., Topol E.J. (2023). Long COVID: Major findings, mechanisms and recommendations. Nat. Rev. Microbiol..

[11] Wong M.C., Huang J., Wong Y.Y., Wong G.L., Yip T.C., Chan R.N., Chau S.W.-H., Ng S.-C., Wing Y.-K., Chan F.K.-L. (2023). Epidemiology, Symptomatology, and Risk Factors for Long COVID Symptoms: Population-Based, Multicenter Study. JMIR Public Health Surveill..

[12] Cazé A.B., Cerqueira-Silva T., Bomfim A.P., de Souza G.L., Azevedo A.C., Brasil M.Q., Santos N.R., Khouri R., Dan J., Bandeira A.C. (2023). Prevalence and risk factors for long COVID after mild disease: A cohort study with a symptomatic control group. J. Glob. Health.

[13] Garg M., Maralakunte M., Garg S., Dhooria S., Sehgal I., Bhalla A.S., Vijayvergiya R., Grover S. (2021). The Conundrum of ’Long-COVID-19’: A Narrative Review. Int. J. Gen. Med..

[14] Azambuja P., Bastos L.S.L., Batista-da-Silva A.A., Ramos G.V., Kurtz P., Dias C.M.C., da Silvag E.P., Aroucag L.E., Soaresh J., Sejvar J.J. (2024). Prevalence, risk factors, and impact of long COVID in a socially vulnerable community in Brazil: A prospective cohort study. Lancet Reg. Health Am..

[15] Hou Y., Gu T., Ni Z., Shi X., Ranney M.L., Mukherjee B. (2025). Global Prevalence of Long COVID, its Subtypes and Risk factors: An Updated Systematic Review and Meta-Analysis. medRxiv.

[16] Beale S., Yavlinsky A., Moncunill G., Fong W.L.E., Nguyen V.G., Kovar J., Hayward A.C., Abubakar I., Aldridge R.W. (2025). Anti-nucleocapsid and anti-spike antibody trajectories in people with post-COVID condition versus acute-only infections: A nested longitudinal case-control study within the Virus Watch prospective cohort. Nat. Commun..

[17] Varnai R., Molnar T., Zavori L., Tőkés-Füzesi M., Illes Z., Kanizsai A., Csecsei P. (2022). Serum Level of Anti-Nucleocapsid, but Not Anti-Spike Antibody, Is Associated with Improvement of Long COVID Symptoms. Vaccines.

[18] di Filippo L., Frara S., Nannipieri F., Cotellessa A., Locatelli M., Rovere Q.P., Giustina A. (2023). Low Vitamin D Levels Are Associated with Long COVID Syndrome in COVID-19 Survivors. J. Clin. Endocrinol. Metab..

[19] Nielsen N.M., Junker T.G., Boelt S.G., Cohen A.S., Munger K.L., Stenager E., Ascherio A., Boding L., Hviid A. (2022). Vitamin D status and severity of COVID-19. Sci. Rep..

[20] Imran M., Zia R., Ali M., Nisar H., Iqbal K., Ali A., Iqbal U., Din S.M., Shahid J., Ahsan A. (2024). Therapeutic role of vitamin D in COVID-19 patients. Clin. Nutr. Open Sci..

[21] Sartini M., Del Puente F., Carbone A., Schinca E., Ottria G., Dupont C., Piccinini C., Oliva M., Cristina M.L. (2024). The Effect of Vitamin D Supplementation Post COVID-19 Infection and Related Outcomes: A Systematic Review and Meta-Analysis. Nutrients.

[22] Rattanawongnara M.R. (2021). Long-term effects of COVID-19 or Long COVID-19. Atrama Magz..

[23] Kaur J., Khare S., Sizar O., Givler A. (2025). Vitamin D Deficiency. StatPearls.

[24] Department of Medical Service (Thailand) (2021). Post-COVID-19 Care or Long COVID for Physicians and Healthcare Workers. https://covid19.dms.go.th.

[25] Wangchalabovorn M., Weerametachai S., Leesri T. (2022). Prevalence of post COVID-19 conditions in SARS-CoV-2-infected patients at 3-month telephone follow-up. Reg. Health Promot. Cent..

[26] Institute of Medical Research and Technology Assessment, Department of Medical Services, Ministry of Public Health (2022). Long-term health impacts of patients with COVID-19 and health service provision guidelines. J. Dep. Med. Serv..

[27] Thompson E.J., Williams D.M., Walker A.J., Mitchell R.E., Niedzwiedz C.L., Yang T.C., Huggins C.F., Kwong A.S.F., Silverwood R.J., Di Gessa G. (2022). Risk factors for long COVID: Analyses of 10 longitudinal studies and electronic health records in the UK. Nat. Commun..

[28] Di Gennaro F., Belati A., Tulone O., Diella L., Fiore Bavaro D., Bonica R., Genna V., Smith L., Trott M., Bruyere O. (2023). Incidence of long COVID-19 in people with previous SARS-CoV2 infection: A systematic review and meta-analysis of 120,970 patients. Intern. Emerg. Med..

[29] Chen C., Haupert S.R., Zimmermann L., Shi X., Fritsche L.G., Mukherjee B. (2022). Global Prevalence of Post-Coronavirus Disease 2019 (COVID-19) Condition or Long COVID: A Meta-Analysis and Systematic Review. J. Infect. Dis..

[30] Chailurkit L.O., Ongphiphadhanakul B., Aekplakorn W. (2023). Update on vitamin D status in sunshine-abundant Thailand, 2019–2020. Nutrition.

[31] Barrea L., Verde L., Grant W.B., Frias-Toral E., Sarno G., Vetrani C., Ceriani F., Garcia-Velasquez E., Contreras-Briceño J., Savastano S. (2022). Vitamin D: A Role Also in Long COVID-19?. Nutrients.

[32] Meltzer D.O., Best T.J., Zhang H., Vokes T., Arora V., Solway J. (2020). Association of Vitamin D Status and Other Clinical Characteristics with COVID-19 Test Results. JAMA Netw. Open..

[33] Ashique S., Gupta K., Gupta G., Mishra N., Singh S.K., Wadhwa S., Gulati M., Dureja H., Zacconi F., Oliver B.G. (2023). Vitamin D-A prominent immunomodulator to prevent COVID-19 infection. Int. J. Rheum. Dis..

[34] Fenercioglu A.K. (2024). The Anti-Inflammatory Roles of Vitamin D for Improving Human Health. Curr. Issues Mol. Biol..

[35] Gombart A.F., Pierre A., Maggini S. (2020). A Review of Micronutrients and the Immune System-Working in Harmony to Reduce the Risk of Infection. Nutrients.

[36] Grant W.B., Lahore H., McDonnell S.L., Baggerly C.A., French C.B., Aliano J.L., Bhattoa H.P. (2020). Evidence that Vitamin D Supplementation Could Reduce Risk of Influenza and COVID-19 Infections and Deaths. Nutrients.

[37] Bigman G. (2025). Diet and nutrition in long COVID: Low vitamin D and clinical trial of magnesium and vitamin D—A comprehensive scoping review. Nutrients.

[38] Grant W.B., Boucher B.J., Cheng R.Z., Pludowski P., Wimalawansa S.J. (2025). Vitamin D and Cardiovascular Health: A Narrative Review of Risk Reduction Evidence. Nutrients.

[39] Cardoso F., Araújo C.A.L., Silva J.R., Guimarães A., Taveiro M.V., Figueiroa J.N., Alves J.G.B. (2024). Vitamin D levels and COVID-19 severe pneumonia: A prospective case-control study. medRxiv.

[40] Guerrero-Romero F., Gamboa-Gómez C.I., Rodríguez-Morán M., Orrante M., Rosales-Galindo E., Cisneros-Ramírez I., Arce-Quiñones M., Orona-Díaz K., Simental-Mendia L.E., Martínez-Aguilar G. (2023). Hypomagnesemia and 25-hydroxyvitamin D deficiency in patients with long COVID. Magnes. Res..

